# A method comparison study of a point-of-care blood gas analyser with a laboratory auto-analyser for the determination of potassium concentrations during hyperkalaemia in patients with kidney disease

**DOI:** 10.11613/BM.2020.030702

**Published:** 2020-08-05

**Authors:** Mogamat-Yazied Chothia, Patricia Kassum, Annalise Zemlin

**Affiliations:** 1 Division of Nephrology, Department of Medicine, Faculty of Medicine and Health Sciences, Stellenbosch University, Cape Town, South Africa; 2 Division of Chemical Pathology, Faculty of Medicine and Health Sciences, Stellenbosch University and National Health Laboratory Service (NHLS), Tygerberg Hospital, Cape Town, South Africa

**Keywords:** point-of-care, emergency department, potassium concentrations, hyperkalaemia

## Abstract

**Introduction:**

Hyperkalaemia is a common electrolyte disorder that may cause life-threatening cardiac arrythmias. We aimed to determine the agreement of potassium concentrations between GEM premier 3500 point-of-care blood gas analyser (POC-BGA) and Roche Cobas 6000 c501 auto-analyser in patients with hyperkalaemia.

**Methods:**

A prospective, cross-sectional study of all consecutive adult patients referred to the Renal Unit with a serum potassium concentration ≥ 5.5 mmol/L was performed. A total of 59 paired venous blood samples were included in the final statistical analysis. Passing-Bablok regression and Bland Altman analysis were used to compare the two methods.

**Results:**

The median laboratory auto-analyser potassium concentration was 6.1 (5.9-7.1) mmol/L as compared to the POC-BGA potassium concentration of 5.7 (5.5-6.8) mmol/L with a mean difference of - 0.43 mmol/L and 95% upper and lower limits of agreement of 0.35 mmol/L and - 1.21 mmol/L, respectively. Regression analysis revealed proportional systematic error. Test for linearity did not indicate significant deviation (P = 0.297).

**Conclusion:**

Although regression analysis indicated proportional systematic error, on Bland Altman analysis, the mean difference appeared to remain relatively constant across the potassium range that was evaluated. Therefore, in patients presenting to the emergency department with a clinical suspicion of hyperkalaemia, POC-BGA potassium concentrations may be considered a surrogate for laboratory auto-analyser measurements once clinicians have been cautioned about this difference.

## Introduction

Potassium is the most abundant cation in the human body and is predominantly confined to the intracellular fluid compartment (ICF) (*1*). Most of the total body potassium (98%) is located within the ICF compartment. This distribution of potassium is the major determinant of the resting membrane potential of cells required for nerve excitation and muscle contraction. Potassium disorders may result in serious cardiac arrhythmias and/or muscle weakness (*2*).

Hyperkalaemia is a very common electrolyte disorder in hospitalised patients (*3*-*5*). The most common risk factors associated with hyperkalaemia include acute and chronic kidney disease, diabetes mellitus, cardiac failure, and drugs that interfere with the renin-angiotensin-aldosterone system (*4*, *6*). As the symptoms and physical findings of hyperkalaemia may be very subtle and nonspecific, the diagnosis of hyperkalaemia must be made by alternative means. Since hyperkalaemia affects cardiac conduction, the electrocardiogram (ECG) is frequently used to identify patients at imminent risk for arrhythmias. This investigation is non-invasive, readily available, and easily performed in the emergency department. Numerous studies have reported that the ECG has poor diagnostic accuracy, regardless of the degree of hyperkalaemia (*7*, *8*). Therefore, direct measurements are required to accurately determine blood potassium concentration.

Two types of blood tests are possible. Serum or plasma potassium concentration can be measured in the laboratory, or whole blood potassium concentration can be determined by point-of-care blood gas analysers (POC-BGA). There may be a delay in the processing of the laboratory samples which may cause a factitious increased potassium concentration as well as delaying the initiation of therapy and therefore POC-BGA is an attractive alternative. It has been recommended that the potassium concentrations on POC-BGA can be reliably used in the emergency department (*9*).

Prompt access to results is crucial in the management of patients with life-threatening but reversible medical conditions such as hyperkalaemia. The reliance on point-of-care devices for clinical decision-making, particularly in the emergency setting, has gained much popularity due to its ease of use, less reliance on technical staff members, and most importantly, a marked reduction in turnaround time (TAT) (*10*, *11*).

Pseudohyperkalaemia is defined as a difference between serum and plasma potassium concentration of greater than 0.3 to 0.4 mmol/L provided that the sample was collected using the correct phlebotomy technique, remained at room temperature, and was analysed within one hour of sample collection. It is frequently encountered during thrombocytosis due to potassium release from platelets during clotting (*12*). Other causes include haemolysis, leukocytosis, pre-analytical errors such as potassium-ethylenediaminetetraacetic acid (K-EDTA) contamination, and other incorrect phlebotomy techniques such as fist clenching, prolonged tourniquet application, as well as delays in sample transport to the laboratory (*13*).

Few relevant studies have reported on the diagnostic accuracy of POC-BGA potassium concentration measurements relative to laboratory measurements, however, the average potassium concentrations reported in these studies were not in the hyperkalaemic range (*14*-*17*). We could only identify two retrospective studies that were performed in patients with hyperkalaemia (*9*, *18*). In view of the conflicting results from previous retrospective studies, we performed a prospective cross-sectional study of patients with hyperkalaemia with the aim to determine the agreement between GEM Premier 3500 (Instrumentation Laboratory, Massachusetts, United States of America) POC-BGA and Roche Cobas 6000 c501 (Roche Diagnostics GmbH, Mannheim, Germany) analyser potassium concentrations.

## Materials and methods

### Subjects

**Figure 1:**
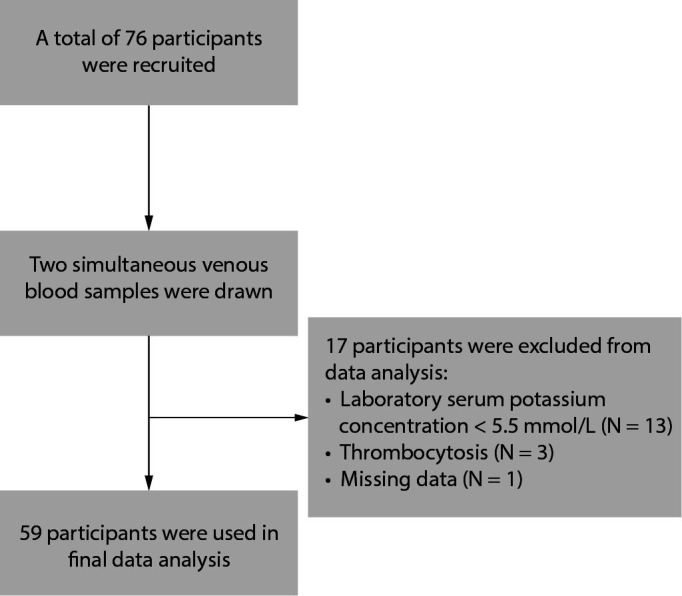
Consort diagram

A cross-sectional study was conducted at Tygerberg Hospital, a 1380 bed tertiary care teaching hospital affiliated to the Faculty of Medicine and Health Sciences of Stellenbosch University. Adult patients (age > 18 years) referred to the Renal Unit with acute kidney injury or chronic kidney disease with hyperkalaemia (≥ 5.5 mmol/L) were included. To reduce the effect of pseudohyperkalaemia, all participants with leukocyte counts > 100 x10^9^/L, platelet counts > 500 x10^9^/L, and haemolysis were excluded, as shown in Figure 1.

Informed consent was obtained. Patients were referred based on prior laboratory auto-analyser measurements. This study was approved by the Human Research Ethics Committee of Stellenbosch University (study number: 7082) and performed according to the Declaration of Helsinki.

### Methods

Paired venous blood samples were obtained from participants over a 6-week period from October to November 2018. Phlebotomy was performed by the same individual using the Joint European Federation of Clinical Chemistry and Laboratory Medicine (EFLM) and the Latin America Confederation of Clinical Biochemistry (COLABIOCLI) recommendations for venous blood sampling (*19*). Paired venous blood samples were drawn simultaneously from the brachial vein and a vein on the dorsum of the hand of the same upper limb because the contralateral upper limb was frequently cannulated with the infusion of intravenous fluids.

For the POC-BGA sample, 2.5 mL of venous blood was drawn with a 23-gauge, 0.65 mm butterfly needle into a prefilled, spray-dried calcium-balanced heparin syringe BD A-line syringe (Becton Dickinson, Wokingham, United Kingdom), capped and turned around gently for one mixing cycle. POC-BGA was performed within five minutes using the GEM Premier 3500 system (Instrumentation Laboratory, Massachusetts, United States of America).

For the laboratory analysis, a sample of 2.5 mL of venous blood was obtained using a closed-loop-system with the help of a vacutainer and bulldog needle into a serum separation tube (BD Vacutainer, Becton Dickinson, Wokingham, United Kingdom). The sample was mixed in the tube by gently inverting the tube for one mixing cycle. The samples were made to stand for at least 30 minutes at room temperature to allow it to clot and thereafter it was centrifuged for 10 minutes at a speed of 3000 revolutions *per* minute. The time of sample collection and time to centrifugation was noted. Samples were hand delivered to a designated laboratory technologist at the chemical pathology laboratory immediately following centrifugation.

The GEM Premier 3500 system (Instrumentation Laboratory, Massachusetts, United States of America) determines the potassium concentration using a potentiometric direct ion selective electrode (ISE). It does not subscribe to an external quality assurance (EQA) program, instead it uses Intelligent Quality Management (iQM). This internal quality assurance system provides continuous monitoring of the analytical process with real-time, automatic error detection. A specific ampoule calibrates the machine for high potassium concentrations (range: 6.2-6.8 mmol/L). During the study period, the measured analytical imprecision was 1% at a nominal target value of 6.8 mmol/L.

In the laboratory, the potassium concentration and the haemolysis index were measured using the Roche Cobas 6000 c501 system (Roche Diagnostics GmbH, Mannheim, Germany). Serum potassium concentration is measured using an indirect ISE method. For a potassium concentration of 6.44 mmol/L, the within-assay coefficient of variation (CV) was 0.5% and the within-laboratory CV was 0.7%. These were within the manufacturer’s recommendation of 0.6% and 0.7%, respectively. Leukocyte and platelet counts were measured using the Siemens ADVIA 2120i haematology system (Siemens Healthcare GmbH, Erlangen, Germany).

The total allowable error (TEa) used for serum potassium was 5.6%, as specified in the Westgard Biological Variation Database (*20*).

### Statistical analysis

Data were analysed using StataCorp. 2019. Stata Statistical Software: Release 16. College Station, TX: StataCorp LLC. The Shapiro-Wilks test was used to test for data normality. Data were expressed as median and interquartile range. Bland Altman analysis and Passing Bablok regression were used to assess the agreement between the two measurement methods. The 95% upper and lower limits of agreement and mean difference was reported for the Bland Altman analysis and the 95% confidence interval (95%CI) for the intercept and the slope were reported for the Passing Bablok regression line. The cumulative sum test (cusum test) was used to calculate linearity between the two methods.

## Results

Seventy-six patients were referred with hyperkalaemia of which 59 were included in the final statistical analysis. Seventeen patients were excluded from the final statistical analysis because the laboratory potassium concentration was < 5.5 mmol/L (N = 13), had thrombocytosis (N = 3), and missing data (N = 1).

The median age was 40 (32-47) years. Thirty participants were female. All participants had kidney disease with 38 having acute kidney injury and 21 with chronic kidney disease.

The median time from sample collection to completion of centrifugation of laboratory samples was 33 (30-36) minutes. The median laboratory potassium concentration was 6.1 (5.9-7.1) mmol/L as compared to the POC-BGA potassium concentration of 5.7 (5.5-6.8) mmol/L with a mean difference of -0.4 mmol/L.

**Figure 2:**
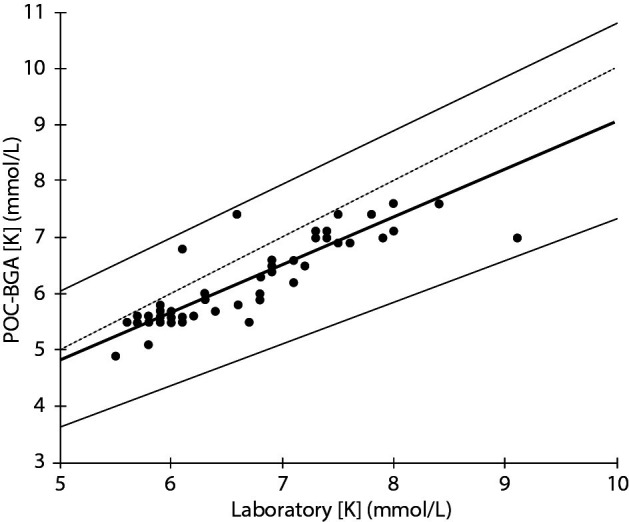
Passing Bablok regression of the two methods for hyperkalaemia. Scatter diagram with regression line and confidence intervals for regression line. Cusum test for linearity did not indicate significant deviation (P = 0.297). Gray lines represent 95% confidence intervals, black line represents the regression line (equation: y = 0.59 + 0.846x, 95%CI for the intercept was -0.09 to 1.26 and for the slope the 95% CI was 0.74 to 0.96) and dotted line represents the line of equality. POC-BGA – point-of-care blood gas analyser. [K] – potassium concentration.

**Figure 3:**
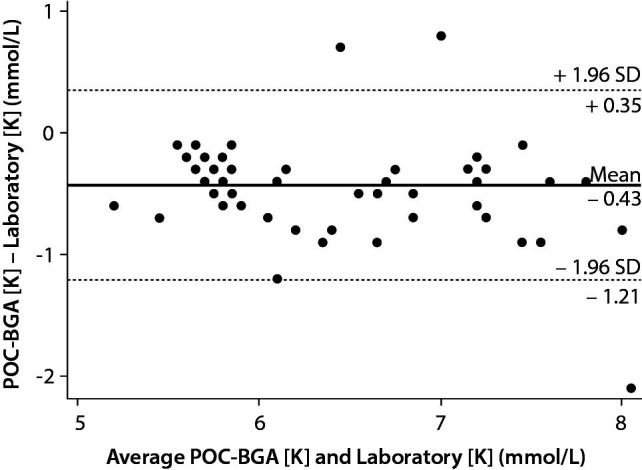
The Bland Altman analysis. Dashed lines indicate upper and lower limits of agreement and solid line indicates the mean difference. [K] – potassium concentration. POC-BGA – point-of-care blood gas analyser. SD – standard deviation.

Figure 2 shows the Passing Bablok regression line with associated 95%CI for the intercept and the slope. The mean difference was - 0.43 mmol/L. The Bland Altman analysis shows the mean difference with the associated 95% upper and lower limits of agreement for the two methods (Figure 3).

## Discussion

To the best of our knowledge, this was the first prospective study to determine the agreement between POC-BGA and laboratory auto-analyser potassium concentrations in patients with hyperkalaemia and kidney disease. A difference in measurements was identified between GEM Premier 3500 POC-BGA and Roche Cobas 6000 c501 auto-analyser and most of the potassium values for the POC-BGA were outside of the TEa of 5.6%. With the visual inspection of the Bland Altman analysis, the mean difference remained relatively constant across the full range of potassium concentrations. However, Passing Bablok regression analysis did not support a constant systematic error between the two methods since the 95%CI for the intercept did not differ significantly from 0; however, the 95%CI for the slope differed significantly from 1, indicating the presence of proportional systematic error (*21*). Although regression analysis indicated proportional error as the potassium concentration rose, we limited our samples to only include potassium concentrations of more than or equal to 5.5 mmol/L. This may have been diminished or absent if the full range of potassium concentrations were evaluated. Previous studies that included samples across a wider range of potassium concentrations did not report any proportional systematic error (*22*-*24*).

Few studies have reported on the agreement between the two methods. A study that examined the agreement between the two methods in patients with chronic kidney disease, reported a difference of -0.4 mmol/L (*16*). Although the latter study had a larger sample size, the median potassium concentrations were < 5.0 mmol/L. Despite this, the reported total error was within the recommended TEa at a threshold value of 5.5 mmol/L. Another study that compared the agreement of numerous analytes, including potassium concentration in critically ill patients, reported a difference of only 0.20 mmol/L, with most of the potassium values within the normal range. Gupta *et al.* compared 112 paired arterial blood gas samples with serum laboratory samples and reported a difference for potassium concentration of -0.14 mmol/L (*25*). The mean serum potassium concentration was within the normal range. In the latter study, after grouping serum potassium concentrations into < 3.5 mmol/L, 3.5 to 5.2 mmol/L and > 5.2 mmol/L, the associated differences were 0.07 mmol/L, 0.26 mmol/L and 0.28 mmol/L, respectively. The only other study to exclusively examine the agreement between the two methods with serum potassium concentrations of more than or equal to 6.0 mmol/L, reported a high mean difference of 0.62 mmol/L (*18*). Therefore, the difference between the two methods appears to be reduced when the whole or normal range of potassium concentrations is evaluated. However, the difference between the two methods increases only when evaluating the higher range of potassium concentrations, as was found in this study and by others (*18*).

The type of blood sample required for analysis may play a role. While POC-BGA requires a whole blood sample, laboratory auto-analysers require a serum sample. To utilise whole blood for POC-BGA, a heparinised sample is required. A study that evaluated the effects of different concentrations of liquid heparin and heparin vacutainers on the measurement of electrolytes reported that liquid formulations had a significant negative bias on measured concentrations of electrolytes (*26*). On the other hand, serum samples may be susceptible to pseudohyperkalaemia. Two factors that contribute to this are the absolute potassium concentrations and the number of platelets (*27*). In serum, during the clotting process, potassium released by platelets cause the serum potassium concentration to rise and can cause a difference between the two methods of approximately 0.3 mmol/L, even when platelet counts are within the normal range (*28*). Drogies *et al.* reported that the difference between the measured plasma and serum potassium concentrations were < 0.1 mmol/L in the presence of hypokalaemia, while it was > 0.5 mmol/L during hyperkalaemia (*27*). Therefore, an additional factor contributing to the difference in measurements identified in our study may be the inclusion of samples with only high potassium concentrations.

This study was the first to prospectively determine the agreement between POC-BGA and laboratory auto-analyser potassium concentrations in patients with hyperkalaemia. As this study was conducted using a standardised phlebotomy technique, hand-delivery of samples to the laboratory and the exclusion of patients with thrombocytosis, and hyperleukocytosis, the difference may be greater in less ideal clinical settings. Also, our results may not be generalisable since the analytic imprecision may differ between laboratories.

In conclusion, the POC-BGA is not a replacement for laboratory auto-analyser measurements but should rather be viewed as complementary, allowing for a rapid response in the emergency department. Although regression analysis indicated a proportional systematic error, on Bland Altman analysis, the mean difference appeared to remain relatively constant across the potassium range that was evaluated. Therefore, in patients presenting to the emergency department with a clinical suspicion of hyperkalaemia, POC-BGA potassium concentrations may be considered a surrogate for laboratory auto-analyser measurements once clinicians have been cautioned about this difference.

## References

[1] Palmer BF Regulation of potassium homeostasis. Clin J Am Soc Nephrol. 2015;10:1050–60. 10.2215/CJN.08580813 24721891 PMC4455213

[2] Medford-Davis L Rafique Z Derangements of potassium. Emergency Medicine Clinics. 2014;32:329–47. 10.1016/j.emc.2013.12.005 24766936

[3] Hayes J Kalantar-Zadeh K Lu JL Turban S Anderson JE Kovesdy CP Association of hypo-and hyperkalemia with disease progression and mortality in males with chronic kidney disease: the role of race. Nephron Clin Pract. 2012;120:c8–16. 10.1159/000329511 22156587 PMC3267990

[4] Sarafidis PA Blacklock R Wood E Rumjon A Simmonds S Fletcher-Rogers J Prevalence and factors associated with hyperkalemia in predialysis patients followed in a low-clearance clinic. Clin J Am Soc Nephrol. 2012;7:1234–41. 10.2215/CJN.01150112 22595825 PMC3408123

[5] Fleet JL Shariff SZ Gandhi S Weir MA Jain AK Garg AX Validity of the International Classification of Diseases 10th revision code for hyperkalaemia in elderly patients at presentation to an emergency department and at hospital admission. BMJ Open. 2012;2:e002011. 10.1136/bmjopen-2012-002011 23274674 PMC4399109

[6] Weir MR Rolfe M Potassium homeostasis and renin-angiotensin-aldosterone system inhibitors. Clin J Am Soc Nephrol. 2010;5:531–48. 10.2215/CJN.07821109 20150448

[7] Montague BT Ouellette JR Buller GK Retrospective review of the frequency of ECG changes in hyperkalemia. Clin J Am Soc Nephrol. 2008;3:324–30. 10.2215/CJN.04611007 18235147 PMC2390954

[8] Wrenn KD Slovis CM Slovis BS The ability of physicians to predict hyperkalemia from the ECG. Ann Emerg Med. 1991;20:1229–32. 10.1016/S0196-0644(05)81476-3 1952310

[9] Jain A Subhan I Joshi M Comparison of the point-of-care blood gas analyzer versus the laboratory auto-analyzer for the measurement of electrolytes. Int J Emerg Med. 2009;2:117–20. 10.1007/s12245-009-0091-1 20157454 PMC2700230

[10] Kendall J Reeves B Clancy M Point of care testing: randomised controlled trial of clinical outcome. BMJ. 1998;316:1052–7. 10.1136/bmj.316.7137.1052 9552905 PMC28507

[11] Parvin CA Lo SF Deuser SM Weaver LG Lewis L Scott M Impact of point-of-care testing on patients’ length of stay in a large emergency department. Clin Chem. 1996;42:711–7. 10.1093/clinchem/42.5.711 8653896

[12] Ranjitkar P Greene DN Baird GS Hoofnagle AN Mathias PC Establishing evidence-based thresholds and laboratory practices to reduce inappropriate treatment of pseudohyperkalemia. Clin Biochem. 2017;50:663–9. 10.1016/j.clinbiochem.2017.03.007 28288853

[13] Zemlin AE Errors in the Extra-Analytical Phases of Clinical Chemistry Laboratory Testing. Indian J Clin Biochem. 2018;33:154–62. 10.1007/s12291-017-0657-2 29651205 PMC5891449

[14] Johnston HL Murphy R Agreement between an arterial blood gas analyser and a venous blood analyser in the measurement of potassium in patients in cardiac arrest. Emerg Med J. 2005;22:269–71. 10.1136/emj.2003.013599 15788827 PMC1726730

[15] Gavala A Myrianthefs P Comparison of point-of-care versus central laboratory measurement of hematocrit, hemoglobin, and electrolyte concentrations. Heart Lung. 2017;46:246–50. 10.1016/j.hrtlng.2017.04.003 28477952

[16] You JS Park YS Chung HS Lee HS Joo Y Park JW Evaluating the utility of rapid point-of-care potassium testing for the early identification of hyperkalemia in patients with chronic kidney disease in the emergency department. Yonsei Med J. 2014;55:1348–53. 10.3349/ymj.2014.55.5.1348 25048495 PMC4108822

[17] Ahn S Kim WY Sohn CH Seo DW Kim W Lim KS Potassium values in cardiac arrest patients measured with a point-of-care blood gas analyzer. Resuscitation. 2011;82:e25–6. 10.1016/j.resuscitation.2011.08.010 21871855

[18] Acikgoz SB Genc AB Sipahi S Yildirim M Cinemre B Tamer A Agreement of serum potassium measured by blood gas and biochemistry analyzer in patients with moderate to severe hyperkalemia. Am J Emerg Med. 2016;34:794–7. 10.1016/j.ajem.2016.01.003 26838187

[19] Simundic A-M Bölenius K Cadamuro J Church S Cornes MP van Dongen-Lases EC Joint EFLM-COLABIOCLI Recommendation for venous blood sampling. Clin Chem Lab Med. 2018;56:2015–38. 10.1515/cclm-2018-0602 30004902

[20] Westgard QC. Desirable Biological Variation Database specifications 2019. Available from: https://www.westgard.com/biodatabase1.htm. Accessed March 19th 2020.

[21] Bilić-Zulle L Comparison of methods: Passing and Bablok regression. Biochem Med (Zagreb). 2011;21:49–52. 10.11613/BM.2011.010 22141206

[22] Novell M Rico N Blondeau P Blasco M Maceira A Bedini JL A novel point-of-care device for blood potassium detection of patients on dialysis: Comparison with a reference method. Nefrologia. 2020;40:363–4. 10.1016/j.nefro.2019.06.002 31627974

[23] Uyanik M Sertoglu E Kayadibi H Tapan S Serdar MA Bilgi C Comparison of blood gas, electrolyte and metabolite results measured with two different blood gas analyzers and a core laboratory analyzer. Scand J Clin Lab Invest. 2015;75:97–105. 10.3109/00365513.2014.981854 25431133

[24] Pant V Tumbapo A Karki B Inter-instrumental comparison for the measurement of electrolytes in patients admitted to the intensive care unit. Int J Gen Med. 2017;10:145–9. 10.2147/IJGM.S135788 28553133 PMC5439724

[25] Gupta S Gupta AK Singh K Verma M Are sodium and potassium results on arterial blood gas analyzer equivalent to those on electrolyte analyzer? Indian J Crit Care Med. 2016;20:233–7. 10.4103/0972-5229.180044 27303138 PMC4906340

[26] Sandler P Goldstein L The effect of different forms of heparin on point-of-care blood gas analysis. S Afr Med J. 2018;108:224–9. 10.7196/SAMJ.2018.v108i3.12626 30004367

[27] Drogies T Ittermann T Lüdemann J Klinke D Kohlmann T Lubenow N Potassium–reference intervals for lithium-heparin plasma and serum from a population-based cohort. Lab Med. 2010;34:39–44. 10.1515/jlm.2010.002

[28] Nijsten MW De Smet B Dofferhoff A Pseudohyperkalemia and platelet counts. N Engl J Med. 1991;325:1107. 10.1056/NEJM199110103251515 1891016

